# Analysis of the EEG Rhythms Based on the Empirical Mode Decomposition During Motor Imagery When Using a Lower-Limb Exoskeleton. A Case Study

**DOI:** 10.3389/fnbot.2020.00048

**Published:** 2020-08-27

**Authors:** Mario Ortiz, Eduardo Iáñez, José L. Contreras-Vidal, José M. Azorín

**Affiliations:** ^1^Brain-Machine Interface Systems Lab, Miguel Hernández University of Elche, Elche, Spain; ^2^Laboratory for Non-Invasive Brain Machine Interfaces, Department of Electrical and Computer Engineering, University of Houston, Houston, TX, United States

**Keywords:** brain-machine interface, frequency analysis, electroencephalography, empirical mode decomposition, exoskeleton, motor imagery

## Abstract

The use of brain-machine interfaces in combination with robotic exoskeletons is usually based on the analysis of the changes in power that some brain rhythms experience during a motion event. However, this variation in power is frequently obtained through frequency filtering and power estimation using the Fourier analysis. This paper explores the decomposition of the brain rhythms based on the Empirical Mode Decomposition, as an alternative for the analysis of electroencephalographic (EEG) signals, due to its adaptive capability to the local oscillations of the data, showcasing it as a viable tool for future BMI algorithms based on motor related events.

## 1. Introduction

A brain-machine interface (BMI) (Rao, 2013) is a system that allows controlling a device through the analysis of the electric biosignals that can be acquired from the brain, with the help of scalp electrodes. Electroencephalographic (EEG) signals are thus acquired in a non-invasive way, for its posterior processing and interpretation by the algorithms associated with the BMI. EEG activity is usually categorized by different rhythms which are associated with certain brain activities and specific frequency bands: activity below 4 Hz is related to Delta band [sleep waves (Amzica and Steriade, 1998) and motion related cortical potentials (Shibasaki and Hallett, 2006)]; the Theta band is usually assessed between 4 and 7 Hz and has been related, in the literature, to response repression (Kirmizi-Alsan et al., 2006); the Alpha band, which oscillates between 8 and 15 Hz, varies when the eyes are closed and it has been used with Beta band (16–31 Hz) for decoding the motor activity (Pfurtscheller et al., 2006); finally, the Gamma band (32–100 Hz) has been used in movement and attentive focus (Rao, 2013; Costa et al., 2016).

BMIs have emerged as a promising tool to rehabilitate or assist people when they are commanding robotic exoskeletons (He et al., 2018). In the case of the control of robotic exoskeletons by means of a BMI, the most common paradigms are based on:

- Movement Related Cortical Potential (MRCP) are time-frequency changes of the signal associated with movements or decision making (Shibasaki and Hallett, 2006). However, their low potential and frequency makes them difficult to detect in a single trial due to the necessity to mitigate artifacts or other mental processes, averaging several events.
- Event related Des/Synchronization (ERD/ERS) is based on the fact that during motor intention there is a previous decrement of the power in alpha and beta bands during the 2 s before the voluntary movement, followed by an increase of the power (Pfurtscheller and Neuper, 1994; Pfurtscheller et al., 1997, 2006). For the clear identification of the ERD/ERS, it is also necessary tp average several trials. This paradigm has also been used during the mental task of motor imagery (MI), as it can produce a similar effect to the real movement (Jeon et al., 2011; Del Castillo et al., 2018).
- Steady-State Visual Evoked Potentials (SSVEPs) are related to the response offered by the brain due to an external stimulus. Its nature is not directly related to the movement or MI, but this response can be used to control an exoskeleton with a BMI (Kwak et al., 2015; Zhang et al., 2015; Gui et al., 2017). Although the potential is not related to a movement action, it can be used to train the user to make a connection between the visual stimulus and the desired robotic action.

In these paradigms, the extraction of features is usually accomplished by the filtering of the frequency bands, associated with the rhythms related to the brain activity being analyzed. Although the features are generally extracted using traditional signal processing filters, there are other alternatives in the literature based on time vs. frequency analysis techniques, such as the wavelet transform (Xu and Song, 2008; Yang et al., 2010; Kant et al., 2019) and the Stockwell transform (Ortiz et al., 2017). However, they have not been used in conjunction with an exoskeleton (He et al., 2018). In the present study, a different approach will be studied using a decomposition algorithm, the Empirical Mode Decomposition (EMD). In this study, the EMD algorithm will be used to detect the variations of the EEG signals during MI tasks in comparison to the relaxed state, when a user is commanding a lower-limb exoskeleton. Due to the oscillatory nature of EEG and the lack of stationary behavior of EEG rhythms, EMD is an interesting algorithm for to use for the analysis of these signals. EMD has been used to detect epilepsy (Martis et al., 2012; Li et al., 2013) or even as an EOG removal technique (Looney et al., 2008), however, even as its utility for EEG analysis has been demonstrated (Rutkowski et al., 2010), its application for developing a BMI to command an exoskeleton has not been explored yet. The results will be compared to those obtained by a Butterworth filter. In addition, the activation of the exoskeleton will be analyzed in the time domain, thanks to the properties of the Hilbert transform.

## 2. Materials and Methods

### 2.1. Experimental Setup

#### 2.1.1. Subjects

Three able-bodied adults voluntarily participated in the study. All the information was given to the subjects before the experiment and they agreed by signing an informed consent form. All procedures were approved by the Institutional Review Board of the University of Houston (USA).

#### 2.1.2. Equipment

Two non-invasive bundles of 32 wet electrodes were used for the EEG acquisition with the help of an actiCap (Brain Products GmbH, Germany) unit. The distribution followed the 10 − 10 international system, reallocating four scalp electrodes around the eyes to assess the ocular artifacts in bipolar configuration (TP10-TP9 for Vertical-EOG and PO10-PO9 for Horizontal-EOG). Reference and ground were placed in the lower lobes of each ear. Data were wirelessly transmitted by a Move transmitter and amplified by two BrainAmp amplifiers (Brain Products GmbH, Germany).

**Figure 1:**
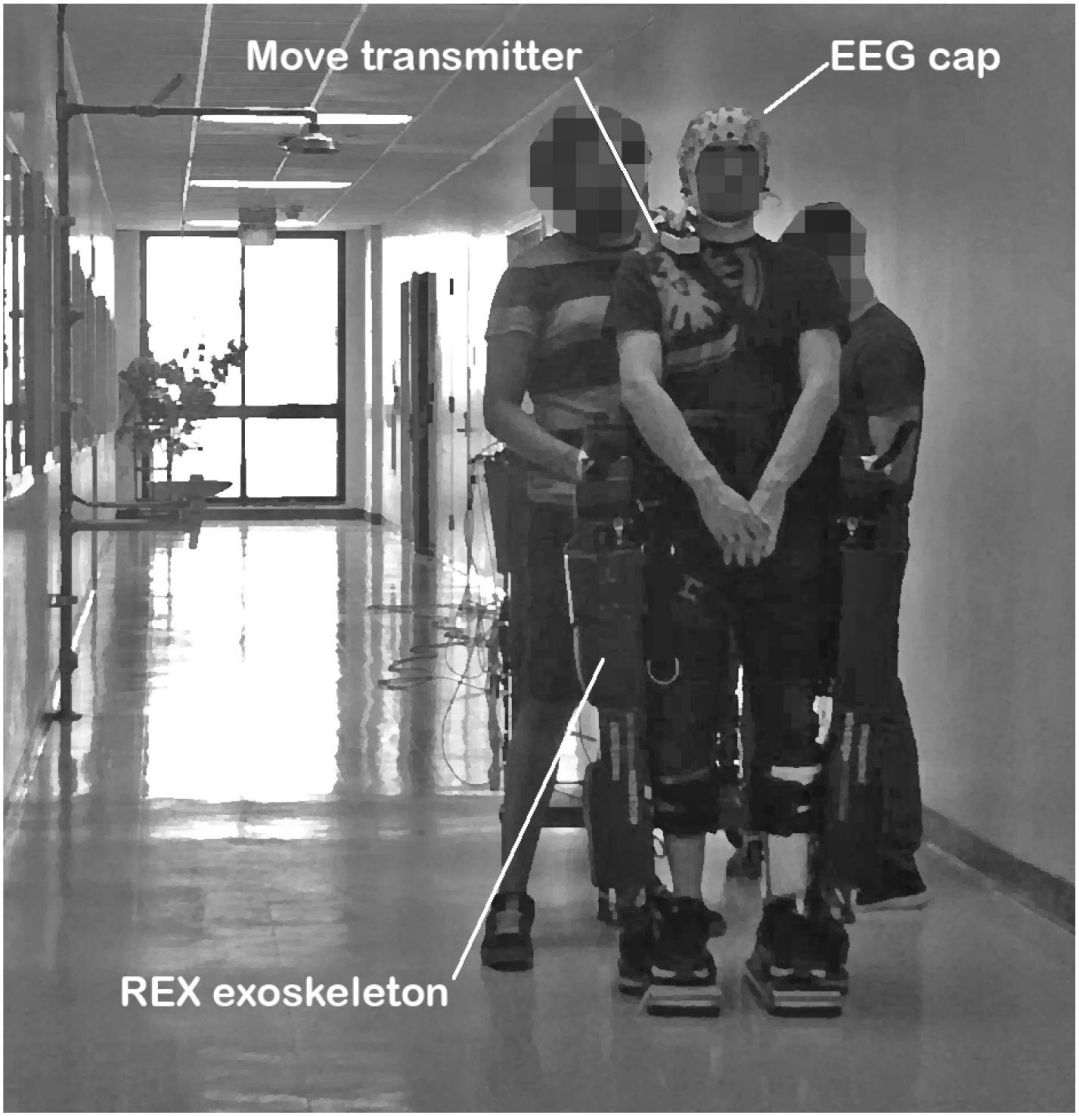
Image of a subject executing one of the experimental trials.

The lower-limb exoskeleton used was the REX (Rex Bionics, New Zealand). Activation commands were sent to the exoskeleton wirelessly, while the status of the exoskeleton was received by the computer through wire serial port communication. The control of the exoskeleton was done by custom firmware developed in Matlab. Figure 1 shows a subject commanding the REX exoskeleton during one of the trials. Although REX can be commanded without external help, two individuals were placed by the sides of the exoskeleton to avoid any potential risk of losing balance.

#### 2.1.3. Protocol

The objective of the paper was to compare the assessment of the EEG variations observed in different rhythms associated with motor imagery tasks when a subject is using a lower-limb exoskeleton. With this aim in mind, the protocol was designed as detailed below.

**Figure 2:**
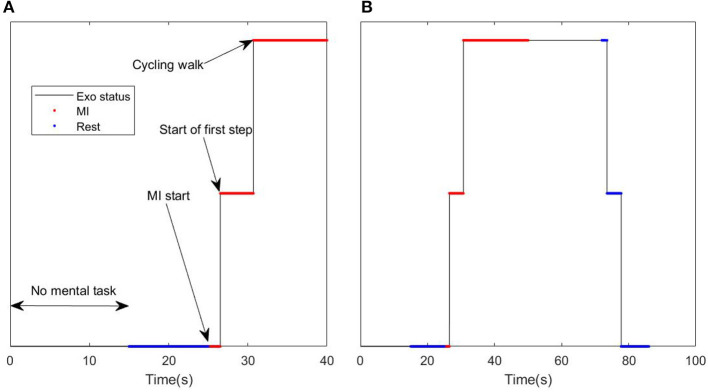
Example of trial: **(A)** Details of the activation command. **(B)** Details of the MI tasks ~20 s (red) and relaxed state event ~10 + 10 s (blue), and moving reverse count (black top).

The whole protocol of a experimental session lasted around 2 h and involved the following parts: (1) adaptation of the exoskeleton to the subject, (2) placing and gelling the 64 electrodes, achieving an impedance value below 20*kΩ* for each electrode, (3) execution of several runs to make sure the subject is comfortable with the REX movement, and finally (4) the fulfillment of the 10 experimental trials. Figure 2 shows the details of an experimental trial, the different events, and the status of the exoskeleton.

Each trial started with 15 s of free mental task to help the subject concentrate in the experiment and to allow enough time to pass for the ocular artifact removal algorithm convergence (Kilicarslan et al., 2016). This period of time was not considered for the analysis, as it was not associated with any mental task. After that, an acoustic signal warned the subject to relax and to try imagine a rest state of no movement. This provided the reference state (bottom blue points) to the MI event (red points) that occurs after the rest event. After 10 s of rest, a new acoustic cue indicated to the subject to focus on the mental task of the movement of their legs (MI start). At the same time, a command was issued to move the exoskeleton. However, as REX has a variable time for activation, the start of the movement lagged for up to 2 s (start of first step point). After the first step was completed (around 5 s after the cue signal) the exoskeleton kept walking at a steady pace (cycling walk) recording the EEG signal for MI assessment (red top). After ~20 s of MI, another cue signal indicated to the subject to start making a reverse count (black top line). This part serves as a control event to be sure that the MI features were not caused by motion artifacts. After another 20 s, a final acoustic cue indicated the final rest event. This final event was not used in this study as it had no associated MI event.

### 2.2. Empirical Mode Decomposition

EMD was developed by Norden E. Huang as a decomposition method to improve the extraction of the instantaneous frequency and amplitude of non-stationary signals (Huang et al., 1998). The algorithm decomposes the original signals in several modes, called intrinsic mode functions (IMF), to provide a better time-frequency representation using the Hilbert transform. Each IMF contains the local oscillation information of the signal, extracted from higher to lower frequencies.

Given a discrete signal *s*(*t*), its EMD can be obtained following the next steps:

1. Assign the signal to a temporal signal *x*(*t*) = *s*(*t*).
2. Find the local extrema of *x*(*t*).
3. Find the maximum envelope *e*_+_(*t*) of *x*(*t*) by passing a natural cubic spline through the local maxima. Similarly, find the minimum envelope, *e*_−_(*t*), with the local minima.
4. Compute an approximation to the local average: *m*(*t*) = (*e*_+_(*t*) + *e*_−_(*t*))/2.
5. Find the proto-mode function *z*_*i*_(*t*) = *x*(*t*) − *m*(*t*).
6. Check whether *z*_*i*_(*t*) is an IMF. An IMF is a wave that satisfies two conditions: the number of extrema and the number of zero crossings may differ by no more than one; and its local average is zero. The threshold used to set this last condition is critical, as a high value can lead to overtraining and a low value can leave components unextracted. Moreover, to avoid the extraction of accidental IMFs, the conditions must be accomplished in at least two or three consecutive iterations.
7. If *z*_*i*_(*t*) is not an IMF, repeat the loop on *z*_*i*_(*t*). If *z*_*i*_(*t*) is an IMF then set *IMF*_*n*_(*t*) = *z*_*i*_(*t*) and begin the process again considering *x*(*t*) = *x*(*t*) − *IMF*_*n*_(*t*), being *n* = 1:*M*.
8. The process stops when the desired limit of IMFs has been achieved (*n* = *M*), or a maximum number of sifting operations have been done *i* = *p*, without the IMF conditions fulfilled. In this case, the residual is considered as $res\left(t\right)=s\left(t\right)-\overset{n=1}{\underset{n=M}{\sum }}IMF_{n}$

Nevertheless, the algorithm is not exempt of drawbacks, such as the difficulty to separate near tones or border effects (Rilling and Flandrin, 2008). Border effects and the time of computation can be a drawback for the EMD employment in real time EEG signals. As processing windows are usually short during online processing, a great part of the data is susceptible to being spoiled by the border effects of a bad envelope calculation during the sifting process. As this research performs an offline analysis of the data, it was exempt of this issue. Border effects were outside the processing windows, as the whole trial was used as input data to the EMD algorithm and not partially processed in epochs. However, its application in real time would require the use of overlapped processing epochs, neglecting the border content. Nevertheless, this effect was studied for the EEG signals of this research. For 1 s epochs overlapped by 0.5 s, the time of processing was around 0.3 s per epoch on average and 40% of the window was spoiled. As the time of computation depends on the hardware, and the border effect depends on the sampling frequency, it is difficult to recommend an epoch length. However, an epoch should be as long and with as short a shifting as the hardware allows for computational times.

### 2.3. Data Analysis

#### 2.3.1. Pre-processing

The data acquisition was done at a 1 kHz sampling frequency. However, in order to obtain a consistent set of IMFs, the data was resampled to 200 Hz. The number of IMFs and the frequency tone associated with them depends on the sampling frequency, so a 1 kHz sampling frequency would not provide relevant tones over the 100 Hz. Resampling to 200 Hz provide tones below 100 Hz.

In addition, the ocular artifact removal algorithm parameters are optimized to work at 100–200 Hz sampling frequencies. The *H*^∞^ algorithm employs the information of the pairs of electrodes TP10-TP9 and PO10-PO9 (Kilicarslan et al., 2016). The optimized parameters used were the following: γ = 1.15, *q* = 10^−10^ and *p*_0_ = 0.5. Finally, a 60*Hz* notch filter was applied to mitigate the network power supply component.

#### 2.3.2. IMF Extraction

**Figure 3:**
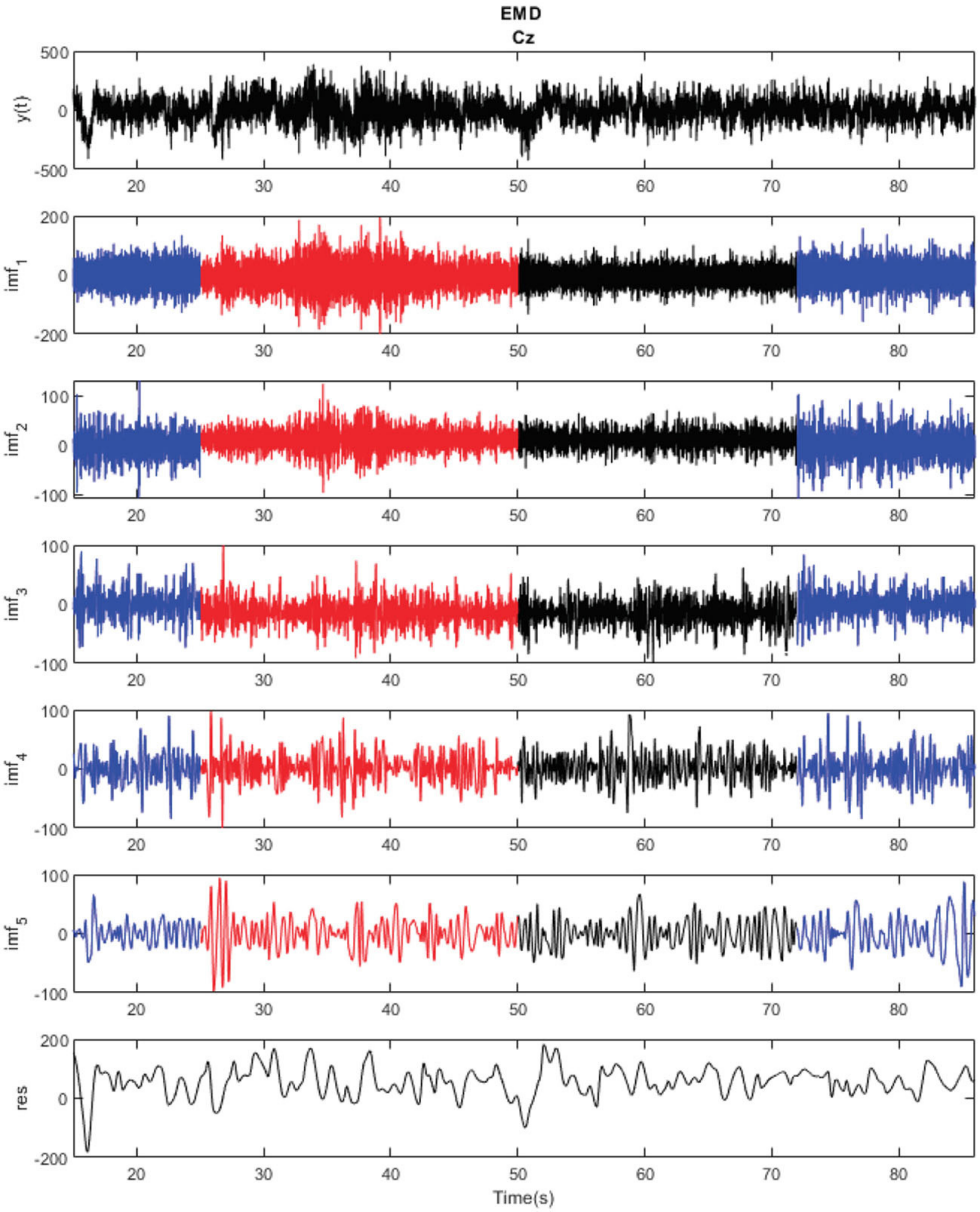
Empirical mode decomposition of one of the trials of subject S2 for Cz electrode. MI periods appear in red and relaxed state periods in blue. Each of the modes corresponds to the local oscillations of the signal in descendent value of frequency.

Once the previous filters were applied, the EMD, as explained in section 2.2, was used to obtain the different modes of the nine electrodes located at the motor cortex: FC1, FCz, FC2, C1, Cz, C2, CP1, CPz, and CP2. Figure 3 shows an example of decomposition of one of the trials for the electrode Cz. Border effects can be seen at the tails of the signal, especially clear in the case of the end of IMF5. In this study, only the first five IMFs were considered (*M* = 5), as they were enough to obtain the EEG rhythms to be analyzed as instantaneous frequency indicators.

Instantaneous frequency (*f*(*t*)) and amplitude (*A*(*t*)) were computed using the Hilbert transform (Huang et al., 1998) of each IMF. Both can be obtained based on the derivative of the instantaneous phase and the module of the analytical function. Given a signal *s*(*t*) = *IMF*_*n*_(*t*), its analytical complex function **z(t)** can be obtained by applying the Hilbert transform (*H*()) as:

(1) $$\begin{array}{l} \text{z(t)}=s\left(t\right)+j\cdot H\left(s\left(t\right)\right)=A\left(t\right)e^{\left(j\phi \left(t\right)\right)} \end{array}$$

obtaining the instantaneous frequency as a function of the derivative of the instantaneous phase:

(2) $$\begin{array}{l} f\left(t\right)=\frac{1}{2\pi }\frac{d\phi \left(t\right)}{dt} \end{array}$$

**Figure 4:**
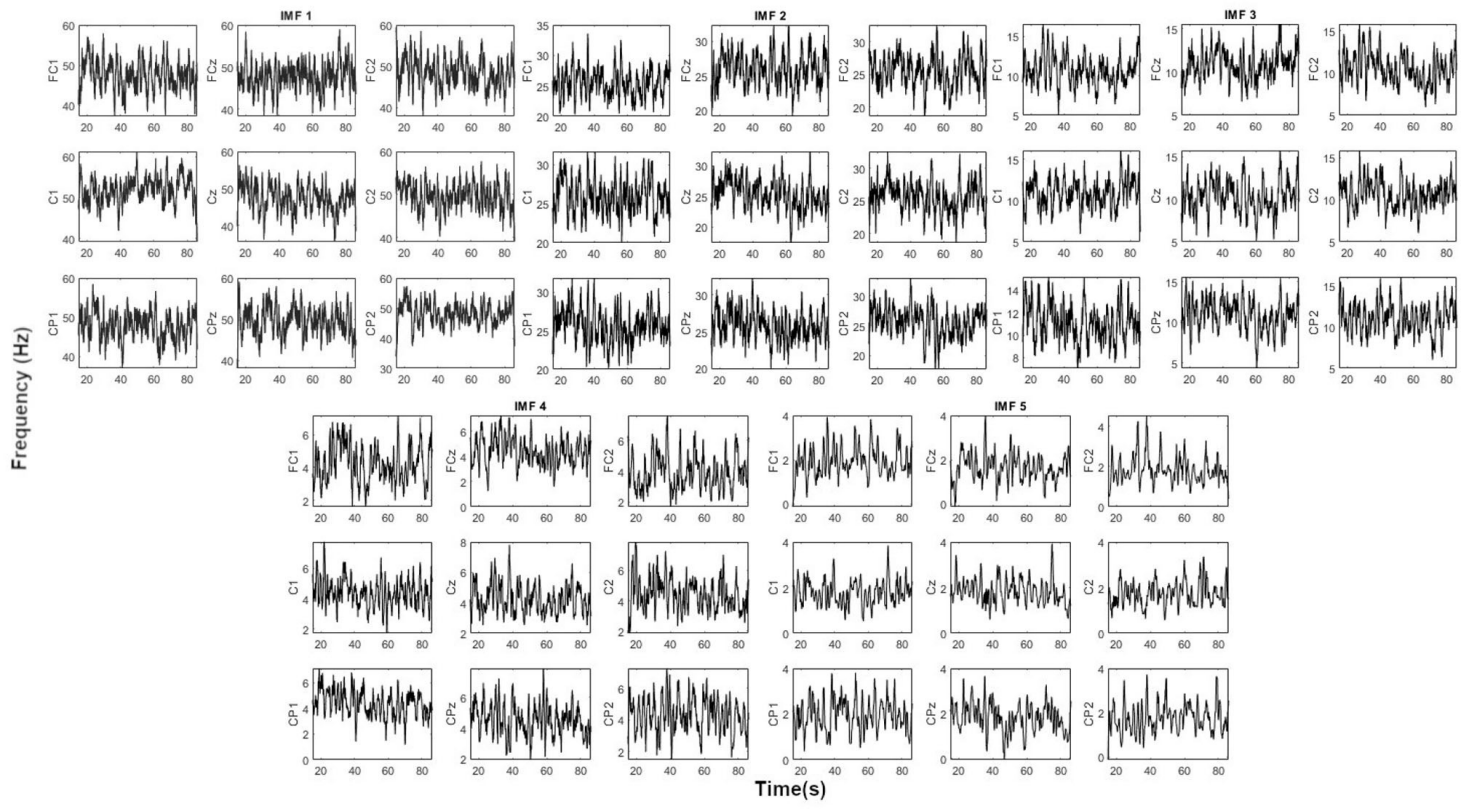
Instantaneous frequency of the first 5 IMFs obtained by EMD of one of the trials of subject S2. Each IMF oscillates within the EEG rhythms: IMF1 (Gamma), IMF2 (Beta), IMF3 (Alpha), IMF4 (Theta), and IMF5 (Delta).

Figure 4 shows the instantaneous frequency for the five IMFs considered. As it can be seen that IMF 1 oscillates in the Gamma band (40–60 Hz), IMF2 in the Beta band (20–30 Hz), IMF3 in the Alpha band (5–15 Hz), IMF4 in the Theta band (2–7 Hz), and IMF5 in the Delta band (<4 Hz). This associates each of the IMFs with a significant EEG rhythm. It is necessary to clarify that the order of the IMF and its relationship with each rhythm can differ depending on the sampling frequency and the possible noise content of the signal. This is one of the reasons why the signals were resampled to 200 Hz in order to have a decomposition similar to the EEG rhythms.

#### 2.3.3. Event Assessment

Two different analyses were carried out. The first analyzed the behavior of the different EEG rhythms during the MI event. In this analysis, the changes of power in the different IMFs were calculated taking the previous rest state as a reference. In order to avoid taking into account evoked potentials, the events considered only the stationary parts of the events. This means that only the power of the IMFs, when the exoskeleton was stopped or during stable walking, was considered (see Figure 2 bottom blue for rest and top red for MI). For a determined start event, the variation of power was assessed for each IMF (*i*) and electrode (*ch*) based on the power of the signal during the MI periods:

(3) $$\begin{array}{l} P_{i,ch}=\overset{t_{MI}=t_{2}}{\underset{t_{MI}=t_{1}}{\sum }}IMF{\left(t\right)}_{i,ch}^{2}/\Delta t \end{array}$$

and the rest periods:

(4) $$\begin{array}{l} R_{i,ch}=\overset{t_{rest}=t_{2}}{\underset{t_{rest}=t_{1}}{\sum }}IMF{\left(t\right)}_{i,ch}^{2}/\Delta t \end{array}$$

as the percentage of variation of the power taking the rest period as reference:

(5) $$\begin{array}{l} \Delta P_{i,ch}\left(\%\right)=\frac{P_{i,ch}-R_{i,ch}}{R_{i,ch}}\cdot 100 \end{array}$$

As Δ*t* = *t*_2_ − *t*_1_ can be different for rest and MI periods, power was divided by it.

In the second analysis, the rhythms were evaluated by its instantaneous variation in time. The instantaneous power of each IMF was computed based on its analytical function as:

(6) $$\begin{array}{l} IP_{i,ch}\left(t\right)=A{\left(t\right)}^{2}=\text{z(t)}\cdot {\text{z}}^{*}\text{(t)} \end{array}$$

## 3. Results

### 3.1. Motor Imagery Analysis (Power Variation)

A statistic analysis was carried out to detect the significant differences for the Δ*P*(%) between the subjects and the electrodes using SPSS (Andy, 2013). Data included the Δ*P*(%) of the five extracted IMFs for each of the three subjects and the nine electrodes during the 10 trials executed, for a total sample size of 270 vectors per IMF. Equation (5) was used for computing the power variation during MI events. The nine electrodes were analyzed to check if there was a similar trend when they were averaged or between the supplementary motor area (SMA), the primary motor cortex (M1), and the pre-motor area (PM): FC1, FCz, FC2, C1, Cz, C2, CP1, CPz, and CP2.

A Shaphiro-Wilks test of normality (S-W) was applied to check for the normality assumption (Ghasemi and Zahediasl, 2012). Averaging the electrodes, S1 and S2 followed a normal distribution for all the IMFs (S1: *D*(10) = [0.885 − 0.0.989], *p* > 0.05; S2: *D*(10) = [0.828 − 0.0.989], *p* > 0.05, appearing statistic in a range for the five IMFs). However, S3 showed a clear deviation from the normal distribution for all the IMFs (S3: *D*(10) = [0.530 − 0.733], *p* < 0.05).

Using the individual electrode Δ*P*(%) output for each IMF as a dependent variable, splitting the data for each subject and electrode, the S-W test results indicated that S1 followed a normal distribution for all the IMFs and electrodes (*D*(10) = [0.870 − 0.986], *p* > 0.05, appearing statistic in a range for the five IMFs and nine electrodes), except IMF1(FC2) [*D*(10) = 0.833, *p* < 0.05], and IMF1(Cz) [*D*(10) = 0.840, *p* < 0.05]. S2 followed a normal distribution for all the IMFs and electrodes (*D*(10) = [0.853 − 0.986], *p* > 0.05), except IMF3(FC1) [*D*(10) = 0.817, *p* < 0.05], IMF3(C1) [*D*(10) = 0.837, *p* < 0.05], and IMF4(C1,Cz) (*D*(10) = [0.708, 0.808], *p* < 0.05). The majority of the electrodes of S3 did not follow a normal distribution (*D*(10) = [0.493 − 0.810], *p* < 0.05, appearing statistic in a range for the five IMFs and nine electrodes), except IMF1(C2) [*D*(10) = 0.911, *p* > 0.05], IMF3(C2) [*D*(10) = 0.865, *p* > 0.05], and IMF5(C2) [*D*(10) = 0.992, *p > 0.05*].

Analyzing Δ*P*(%) of the IMFs as dependent variables for each subject without splitting the data by electrode, the S-W test indicated that S1 followed a normal distribution for all the IMFs with only a small deviation from normality in the Q-Q plot for IMF1 [*D*(90) = 0.919, *p* < 0.05] and a small asymmetry in the histogram toward the high tail. S-W test for S2 indicated a deviation from the normal distribution for IMF1-4 (*D*(90) = [0.947 − 0.972], *p* < 0.05) with only a small skewness (S-shape) in the Q-Q plots. S3 distribution was not normal with a clear double peak shape for all the IMFs in the histograms (*D*(90) = [0.560 − 0.734], *p* < 0.05) and a clear kurtosis deviation in the Q-Q plots, which indicated different performance in some of the trials. As normality could not be assured for all the subjects and electrodes, data were analyzed using non-parametric tests to compare the subject and electrode dependency.

For the averaged electrodes, using the Kruskal-Wallis (K-W) test, the IMF output was significantly affected by the subject (*H*(2) = [9.757, 11.027, 10.934, 17.559, 8.565], *p* < 0.05, appearing in the data for each IMF). The Mann-Whitney test was used to analyze the differences between the subjects. A Bonferroni correction was applied and so all effects are reported at a 0.0167 level of significance. S1 results were significantly different in comparison to S2 for IMFs1-5 (*U* = [14, 17, 12, 0, 15], *r* = [−0.61, −0.56, −0.64, −0.84, −0.59]), and S3 for IMFs1-4 (*U* = [17, 14, 15, 10], *r* = [−0.56, −061, −0.59, −0.68]), but similar for IMF5 (*U* = −1.134, *r* = −0.25). S2 had behaved similarly to S3 for all the IMFs (*U* = [34, 25, 33, 28, 22], *r* = [−0.27, −0.42, −0.29, −0.37, −0.47]).

**Figure 5:**
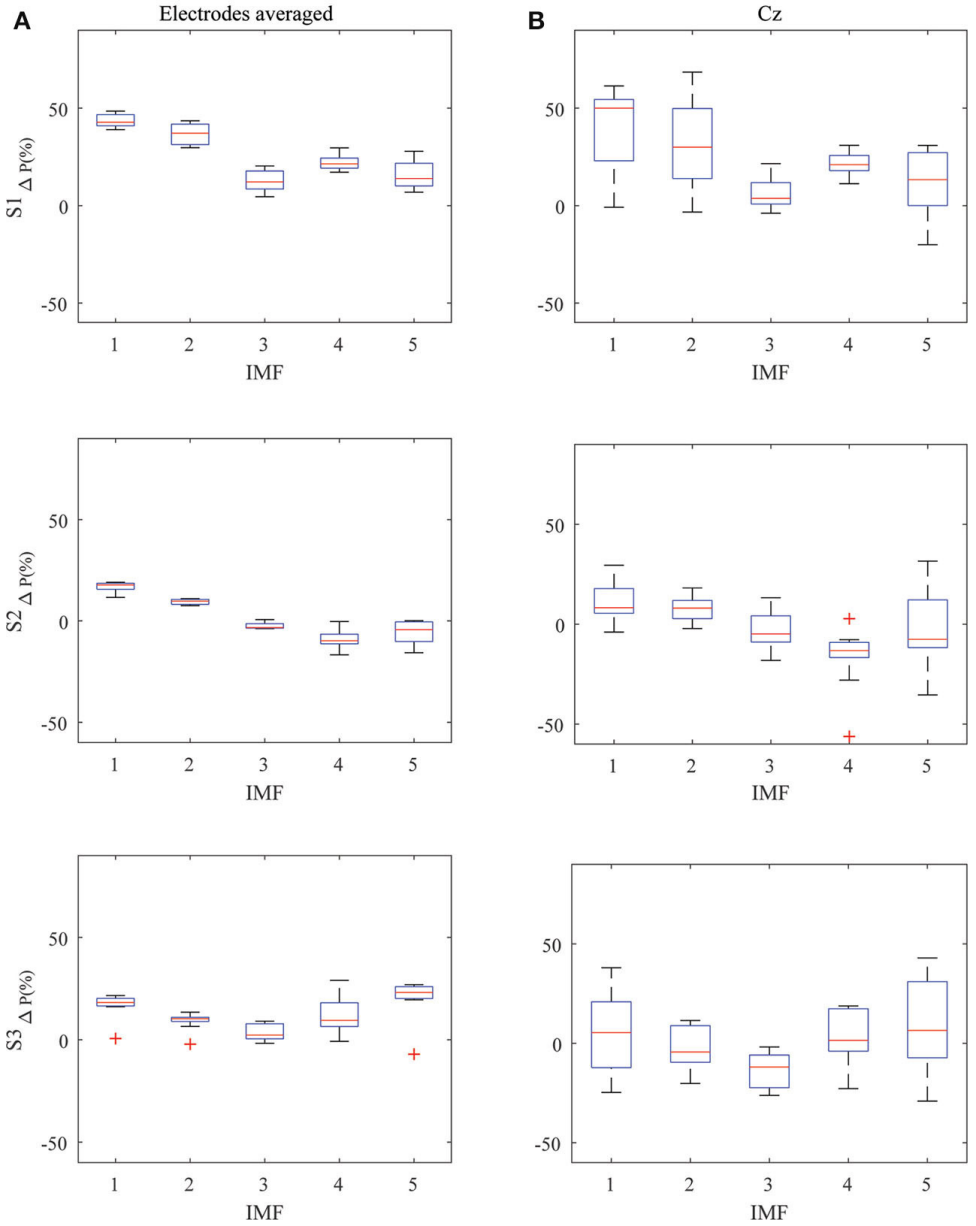
Boxplot of the variation of power of the MI tasks related to the relaxed state: **(A)** Left image shows the variation of power for the nine electrodes averaged through the 10 trials placed in the motor cortex zone. **(B)** Left one shows it for the electrode Cz for the individual 10 single trials.

Analyzing each subject individually, the K-W test indicated that there were no differences between the electrodes for the five IMFs of all the subjects: S1 (*H*(8) = [3.721, 3.279, 10.252, 6.226, 7.732], *p* > 0.05), S2 (*H*(8) = [3.174, 2.183, 2.153, 6.357, 3.910], *p* > 0.05), and S3 (*H*(8) = [1.496, 0.501, 6.790, 11.344, 4.984], *p* > 0.05). This means that the electrodes behaved in a similar way for a given IMF and subject. This matches the boxplot representation. The left part of Figure 5 shows the boxplots of the power variation for the average of the nine electrodes during the 10 trials accomplished by a subject, while the right part shows the same data for the Cz electrode. The associated descriptive statistics to the boxplots can be seen in Table 1 for all the electrodes and its average, and particularized for the Cz electrode in Table 2. As can be seen, if the electrodes were averaged through the 10 trials, there was a similar trend for the three subjects, with a clear increment of the power, especially for high frequency IMFs associated with gamma (IMF1) and beta bands (IMF2). However, IMFs3-5 (alpha, theta, and delta bands) showed a more erratic behavior. Focusing our attention on one of the most representative electrodes, Cz (see right part of Figure 5), its boxplot follows the same trend than the average. However, the deviation, as can be seen in the boxplot and Table 2, is higher, which indicates the difficulty to see this pattern in a single trial without averaging the electrodes.

**Table 1:** Descriptive statistics of the variation of power for the first 5 IMFs averaged through the 10 trials.

**Electrode**	**Subject**	**IMF1**	**IMF2**	**IMF3**	**IMF4**	**IMF5**
	S1	41,6	33.1	18.3	29.7	27.9
FC1	S2	17.7	11.0	−0.9	−9.4	−12.4
	S3	17.7	10.9	2.3	6.6	23.1
	S1	39.0	29.7	4.5	24.1	6.9
FCz	S2	18.4	9.4	−3.8	−9.9	−9.5
	S3	18.2	6.5	4.6	6.1	25.9
	S1	40.0	31.6	11.7	25.3	17.8
FC2	S2	18.2	9.8	−3.8	−13.2	−15.7
	S3	20.0	10.7	9.0	8.7	25.1
	S1	42.8	39.1	17.5	20.0	21.7
C1	S2	17.0	10.9	−2.6	−4.7	−0.3
	S3	16.1	9.8	1.2	9.5	21.6
	S1	41.3	30.6	6.2	21.4	10.6
Cz	S2	11.6	7.6	−3.4	−16.7	−3.4
	S3	16.6	11.0	−1.7	18.4	20.4
	S1	44.2	37.2	9.3	19.2	13.9
C2	S2	15.7	7.5	−3.5	−9.8	−4.9
	S3	0.6	−2.2	−0.2	−0.8	−7.1
	S1	46.4	41.9	20.4	17.2	21.9
CP1	S2	19.1	10.0	−3.3	−0.3	−4.3
	S3	20.9	9.8	0.6	17.9	26.1
	S1	47.6	43.5	12.1	19.2	8.6
CPz	S2	19.0	8.4	0.7	−7.2	0.1
	S3	19.8	10.2	8.7	14.1	19.5
	S1	48.5	41.8	17.6	21.4	11.7
CP2	S2	15.4	10.5	−1.5	−10.7	−0.5
	S3	21.6	13.5	7.5	29.0	26.9
	S1	43.5 ± 3.4	36.5 ± 5.4	13.1 ± 5.7	21.9 ± 3.8	15.7 ± 7.1
Average	S2	16.9 ± 2.4	9.5 ± 1.3	−2.5 ± 1.6	−9.1 ± 4.7	−5.7 ± 5.7
	S3	16.8 ± 6.4	8.9 ± 4.5	3.6 ± 4.0	12.2 ± 8.8	20.2 ± 10,5

*The table shows the ΔP(%) for each electrode and the averaged value ± the standard variation*.

**Table 2:** Descriptive statistics of the variation of power for Cz electrode for the first 5 IMFs during the ten single trials.

	**IMF 1**	**IMF 2**	**IMF 3**	**IMF 4**	**IMF 5**
S1	41.3 ± 21.0	30.5 ± 25.6	6.2 ± 15.1	21.4 ± 12.3	2.6 ± 21.2
S2	11.6 ± 12.9	7.6 ± 8.5	−3.4 ± 7.7	−16.7 ± 10.3	−3.4 ± 31.2
S3	16.6 ± 43.7	11.0 ± 52.9	−1.7 ± 52.9	18.4 ± 53.5	20.4 ± 55.6

*Data show the averaged ΔP(%) ± the standard variation*.

The comparison of the averaged Δ*P*(%) calculated using EMD and the components extracted from the signal by a second order Butterworth filter, show that the results were similar (Table 3). The table shows, using bold text, the methods with a higher increment for Δ*P*(%) in the first two IMFs.

**Table 3:** Comparison between the averaged Δ*P*(%) calculated using EMD and signal filtering by a second order Butterworth filter.

**Method**	**Signal/band**	**S1**	**S2**	**S3**
EMD	IMF1	**43.5** **±** **3.4**	16.9 ± 2.4	16.8 ± 6.4
Freq. Filter	32–49 Hz	42.4 ± 4.6	**31.3** **±** **1.1**	**18.0** **±** **7.2**
EMD	IMF2	**36.5** **±** **5.4**	9.5 ± 1.3	**8.9** **±** **4.5**
Freq. Filter	16–31 Hz	36.3 ± 3.2	**17.6** **±** **0.6**	5.9 ± 3.5
EMD	IMF3	13.1 ± 5.7	−2.5 ± 1.6	3.6 ± 4.0
Freq. Filter	8–15 Hz	32.3 ± 3.0	6.4 ± 1.7	6.1 ± 3.9
EMD	IMF4	21.9 ± 3.8	−9.1 ± 4.7	12.2 ± 8.8
Freq. Filter	4–7 Hz	4.5 ± 4.0	−6.3 ± 1.7	8.1 ± 5.3
EMD	IMF5	15.7 ± 7.1	−5.7 ± 5.7	20.2 ± 10.5
Freq. Filter	1–4 Hz	18.4 ± 4.2	−14.7 ± 1.9	11.5 ± 7.9

*The highest increments for the high frequency components appear in bold*.

### 3.2. Gait Intention Analysis (Instantaneous Power)

In order to study the transient when a subject focuses on the motion intention, an additional study was carried out. The ERD/ERS phenomenon indicates that for certain subjects, the motor intention involves a desynchronization of the mu band (8–12 Hz) followed by a posterior synchronization (Pfurtscheller et al., 2006). Although the phenomenon can be observed in a single trial, it usually requires averaging several trials. Moreover, the effect is also more difficult to notice in foot imagery than in hands. In this study, EMD is proposed as a technique to evaluate the desynchronization and posterior synchronization of the signal during the motion intention. As literature focuses its attention on the mu band, the IMF under study was IMF3, as its instantaneous frequency oscillated around 10 Hz (see Figure 4 IMF3).

**Figure 6:**
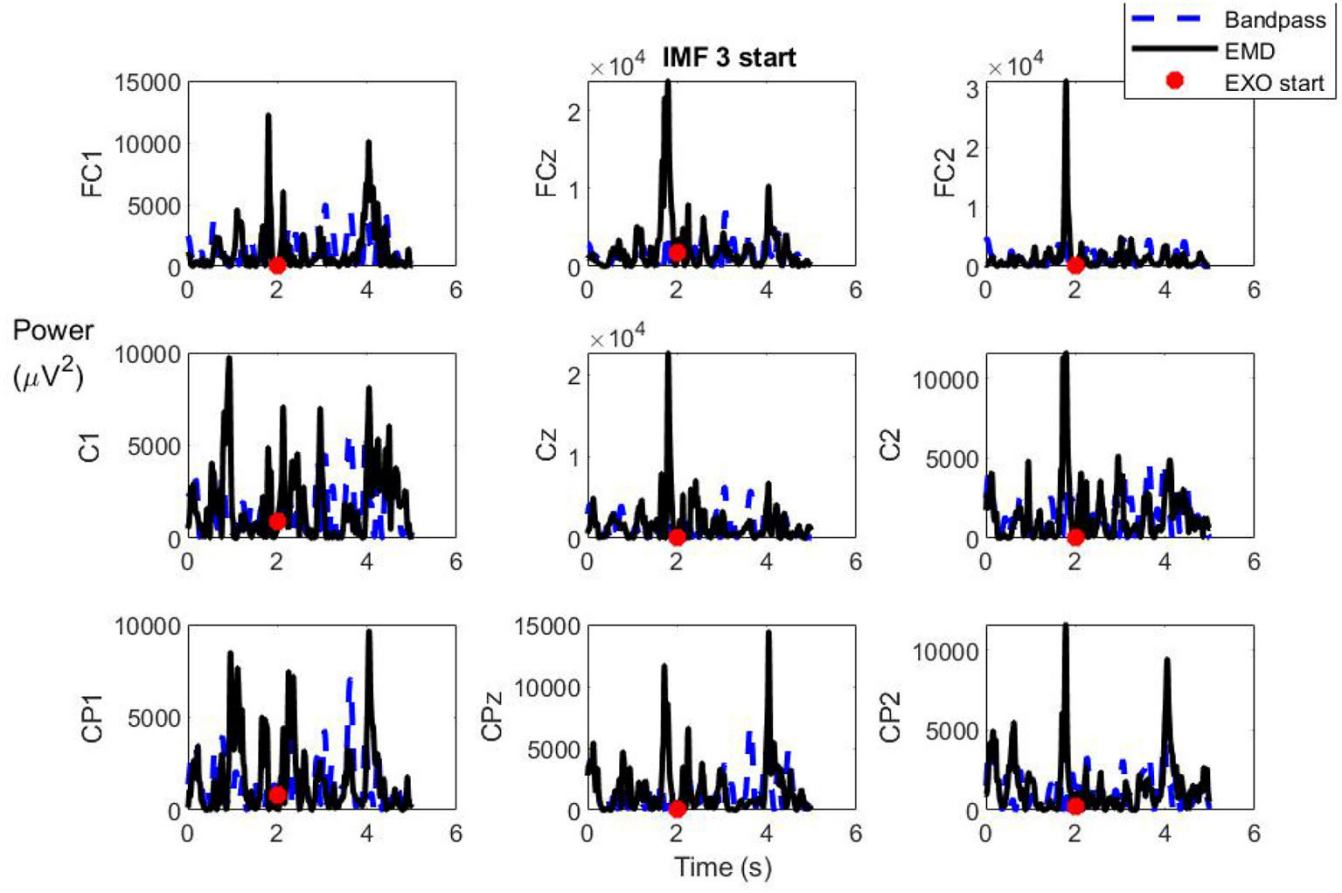
Instantaneous power of IMF3 after the acoustic cue for MI (black line) for trial 1 of subject S1. The value is compared with the instantaneous power of the signal filtered by a Butterworth filter (5–15 Hz). The peak response is higher using the IMF. REX start of movement is represented by a red spot.

With this purpose in mind, the instantaneous power of IMF3 was assessed as indicated by equation 6. Figure 6 shows the instantaneous power during the 5 s after the acoustic cue was issued (*t* = 0) for one of the trials (black line). A red spot indicates the real start of the REX movement, which indicates that the previous time to the red spot is a MI period with no movement, exempt for this reason of any possible motion artifacts. The instantaneous power was compared to the one obtained by only using a second order Butterworth bandpass filter to the signal between 8 and 15 Hz (blue line). Upon inspection of the figure, it can be seen that there are several peaks during the motor imagery period previous to the activation of the exoskeleton. For several electrodes there is a first peak around 1 s before the movement and another one just before the movement. As can be seen, the peak that appears just before the start of the movement is especially clear, using the IMF power (black line), compared to that of the filtered signal (blue line). This indicates that there is an increment of power around 1 s after the acoustic cue, followed by a decrement and a posterior increment just before the real movement starts, which could be related to the ERD/ERS phenomenon. However, the peak magnitudes are variable, which makes it difficult to quantify the phenomenon in a single trial.

Table 4 shows a comparison of both methods for the time at which the maximum peak of the instantaneous power is computed in the five s after the acoustic cue for the MI event is issued. The mean time shows the averaged time for the 10 trials accomplished by each subject per electrode. A negative time indicates that the peak achieved was average for a channel before the REX activation. According to K-W test, the subjects showed no significant differences when using EMD [*H*(2) = 5.42, *p* > 0.05], while they did for the frequency filter band [*H*(2) = 27.13, *p* < 0.001]. As Table 4 shows, a negative time was achieved for the average of the 10 trials in all the subjects for the electrodes C1, CPz, Cz, and FC2 when using the EMD method. The filtered signal showed a higher dispersion for the times, detecting the intention later for the majority of the electrodes.

**Table 4:** Comparison between IMF3 and the signal filtered between 8 and 15 Hz for the detection time of gait intention.

				**95% Confidence interval**	
**Electrode**	**Subject**	**Method**	**Mean time (s)**	**Lower bound**	**Upper bound**
C1(EMD)	S1	**EMD**	**−0.130**	−0.691	0.432
		**Freq. Filter**	**−0.469**	−1.044	0.106
	S2	**EMD**	**−0.453**	−1.015	0.109
		Freq. Filter	0.009	−0.566	0.584
	S3	**EMD**	**−0.503**	−1.065	0.059
		**Freq. Filter**	**−0.388**	−0.963	0.187
C2	S1	**EMD**	**−0.152**	−0.714	0.410
		**Freq. Filter**	**−0.016**	−0.592	0.559
	S2	EMD	0.044	−0.518	0.605
		**Freq. Filter**	**−0.463**	−1.038	0.113
	S3	EMD	0.080	−0.482	0.642
		Freq. Filter	0.358	−0.217	0.933
CP1	S1	EMD	0.019	−0.542	0.581
		Freq. Filter	0.180	−0.396	0.755
	S2	**EMD**	**−0.025**	−0.587	0.537
		**Freq. Filter**	**−0.283**	−0.858	0.292
	S3	**EMD**	**−0.489**	−1.051	0.073
		**Freq. Filter**	**−0.832**	−1.407	−0.257
CP2	S1	EMD	0.127	−0.435	0.688
		Freq. Filter	0.183	−0.392	0.758
	S2	**EMD**	**−0.568**	−1.129	−0.006
		**Freq. Filter**	**−0.238**	−0.813	0.338
	S3	**EMD**	**−0.226**	−0.787	0.336
		**Freq. Filter**	**−0.516**	−1.091	0.059
CPz (EMD)	S1	**EMD**	**−0.128**	−0.689	0.434
		Freq. Filter	0.250	−0.325	0.825
	S2	**EMD**	**−0.728**	−1.289	−0.166
		**Freq. Filter**	**−0.538**	−1.113	0.037
	S3	**EMD**	**−0.242**	−0.804	0.320
		**Freq. Filter**	**−0.730**	−1.305	−0.154
Cz (EMD)	S1	**EMD**	**−0.162**	−0.724	0.400
		Freq. Filter	0.120	−0.455	0.695
	S2	**EMD**	**−0.449**	−1.011	0.113
		Freq. Filter	0.177	−0.398	0.752
	S3	**EMD**	**−0.260**	−0.822	0.302
		**Freq. Filter**	**−0.657**	−1.232	−0.081
FC1	S1	**EMD**	**−0.077**	−0.639	0.485
		Freq. Filter	0.264	−0.312	0.839
	S2	EMD	0.139	−0.423	0.700
		Freq. Filter	0.234	−0.342	0.809
	S3	EMD	0.069	−0.493	0.630
		**Freq. Filter**	**−0.549**	−1.124	0.027
FC2 (EMD)	S1	**EMD**	**−0.627**	−1.188	−0.065
		Freq. Filter	0.384	−0.191	0.959
	S2	**EMD**	**−0.051**	−0.613	0.511
		Freq. Filter	0.590	0.014	1.165
	S3	**EMD**	**−0.123**	−0.685	0.439
		**Freq. Filter**	**−0.696**	−1.271	−0.121
FCz	S1	EMD Freq. Filter	0.126 0.588	−0.436 0.013	0.687 1.163
	S2	**EMD**	**−0.221**	−0.782	0.341
		**Freq. Filter**	**−0.404**	−0.979	0.172
	S3	**EMD**	**−0.109**	−0.671	0.452
		**Freq. Filter**	**−0.817**	−1.392	−0.242
Avg.	S1	**EMD**	**−0.111** **±** **0.224**		
		Freq. Filter	0.165 ± 0.292		
	S2	**EMD**	**−0.257** **±** **0.304**		
		**Freq. Filter**	**−0.102** **±** **0.378**		
	S3	**EMD**	**−0.200** **±** **0.208**		
		**Freq. Filter**	**−0.536** **±** **0.365**		

*The maximum peak is computed for each electrode within the 5 s after the MI acoustic cue is issued. Zero reference is marked by the instant the exoskeleton starts moving. A negative time indicates that the detection is accomplished before the exoskeleton starts moving. The table shows the averaged time for the ten trials of each subject and its 95% confidence interval. Electrodes with negative times are in bold and methods with negative times for all the subjects appear between brackets*.

**Figure 7:**
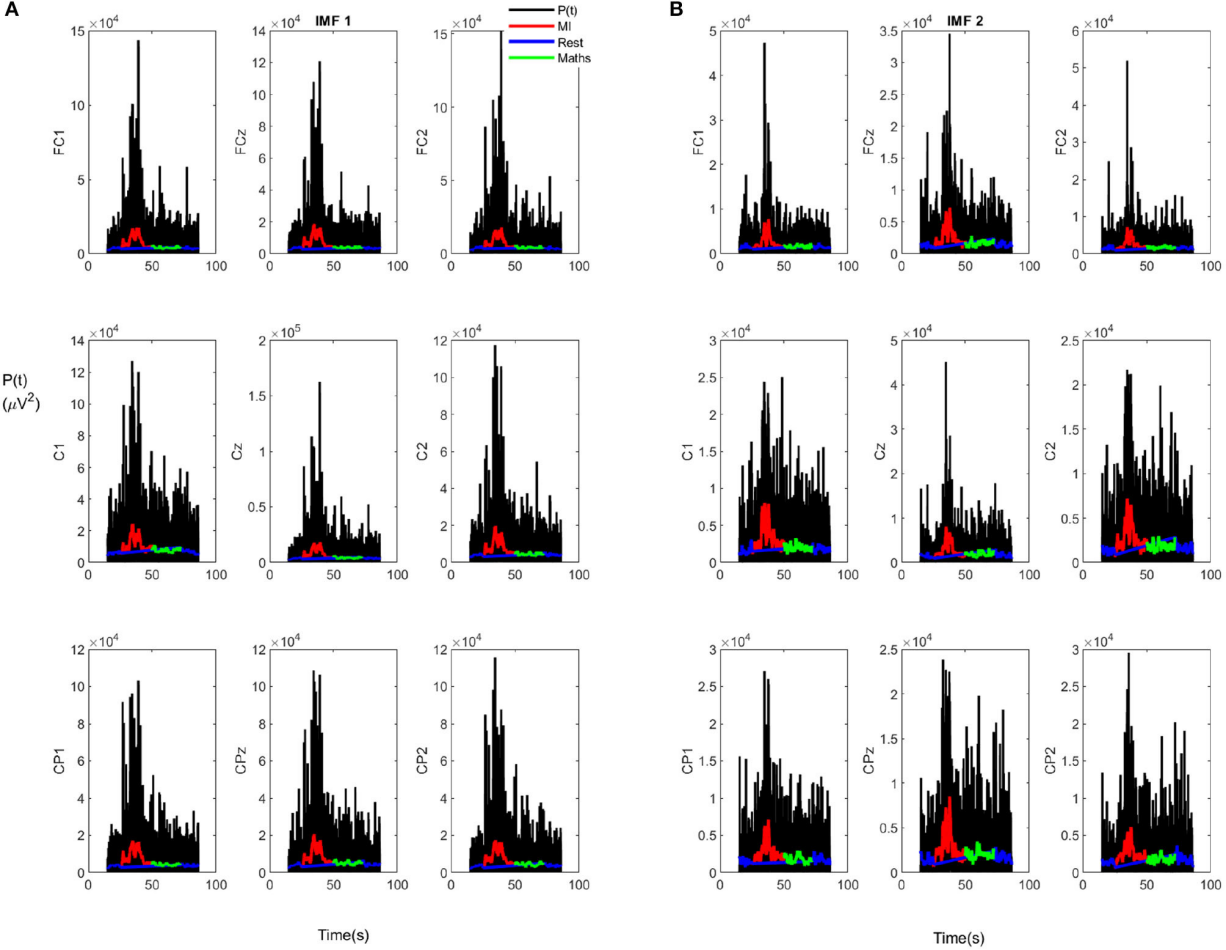
Evolution of the instantaneous power for trial 1 of subject S2: **(A)** IMF1. **(B)** IMF2.

Furthermore, Figure 6 shows that there are also several peaks for certain electrodes after the exoskeleton started moving. To check the evolution and magnitude of these peaks and its possible relationship with the statistical results of section 3.1, the instantaneous power is represented for the full trial in Figure 7. This was limited to IMF1 and IMF2, as these IMFs were the ones that showed a clearer increment of power during MI in Table 2. To see the behavior in a clearer way, the instantaneous power was averaged with a moving mean of 1 s. Figure 7 shows this averaged value for the MI (red), rest (blue), and the control period of the reverse count (green) events. Upon inspection of Figure 7, it is evident that the MI periods show a clear increment in rest periods. In addition, they also show an increment in reverse count operations, which indicates that the increment is not related to any possible motion or exoskeleton artifact, since during the reverse count periods the exoskeleton was moving in the same way than during the MI events. In fact, reverse count periods show a similar power to rest periods.

## 4. Discussion

By analyzing the results, it has been demonstrated that EMD can be a useful tool in characterizing the EEG rhythms during MI events associated with a BMI operating an exoskeleton.

Regarding motor imagery, an increment of power was noticed, not only in average for the MI vs. rest periods, but also in comparison to an alternative mental task during movement (reverse count) in the case of the high frequency IMFs. The power for the IMFs associated with these IMFs showed a different pattern only for the MI events, while showing a similar one for the rest periods. This is consistent with the literature on gamma power increment during motion (Seeber et al., 2014). This behavior could be explained by the attention focus to gait during these periods (Costa-García et al., 2019), which could be useful for the future development of a BMI based on high frequency rhythms assessing the attention to gait. On the other hand, lower frequency IMFs showed a more inconsistent behavior during MI tasks. This can be explained by the nature of EMD, as low frequency IMFs are more susceptible to over-training problems. In addition, in the case of a real-time application, shorter windows of computation could make it more difficult to obtain a consistent result using these IMFs due to possible border effects. This advise against the use of high order IMFs (low frequency ones), at least for BMI control, even as the residual captures the low frequency oscillations of the EEG channel in a precise way (see Figure 3). Furthermore, the statistical analysis revealed that the results were dependant on the subject. However, given a determined subject and IMF, they have a similar behavior for the nine electrodes considered. Nevertheless, the high deviation of the Δ*P*(%) per individual electrode makes its use in a single trial difficult, without averaging the nine electrodes through the trials.

Regarding motor intention, the analysis revealed important facts. As Figure 6 shows, motor intention analyzed by the IMF associated with alpha band followed a pattern similar to the one explained by the ERD/ERS (Pfurtscheller et al., 2006; Jeon et al., 2011) in a single trial. The effect was more noticeable than when using a frequency filter. However, it requires a processing window of at least 4 s, which could compromise the BMI response in real time, as processing would delay the command and increase the computational times. In addition, the magnitude of the peaks was variable, which makes computing them in a significant stable base, or to estimate a statistical trend to be used in a BMI algorithm, difficult, even as statistically the maximum peak is usually achieved during the first 5 s before the actual start of movement.

The results obtained for the Δ*P*(%) were similar to the ones obtained for the filtered signal, but clearer for the motor intention despite its higher computational load. Nevertheless, EMD could be an interesting alternative for the assessment of ERD/ERS, but would require further investigation and an experimental protocol specifically designed for its study. Future research will explore the viability of using this paradigm for the detection of motor intention through the analysis of moving epochs and the usability of high frequency features, extracted with EMD or other time-frequency algorithms, for a BMI that commands an exoskeleton in real time.

## Data Availability Statement

The datasets presented in this article are not readily available because of University of Houston IRB restrictions. Requests to access the datasets should be directed to JC-V (jlcontreras-vidal@uh.edu).

## Ethics Statement

The studies involving human participants were reviewed and approved by Institutional Review Board of the University of Houston (USA). The patients/participants provided their written informed consent to participate in this study. Written informed consent was obtained from the individuals for the publication of any potentially identifiable images or data included in this article.

## Author Contributions

MO and JA: conceptualization. MO: methodology, validation, formal analysis, investigation, writing—original draft preparation, and visualization. MO and EI: software and data curation. JC-V: resources. MO, EI, JC-V, and JA: writing—review and editing. JC-V and JA: supervision and project administration. MO, JC-V, and JA: funding acquisition. All authors contributed to the article and approved the submitted version.

## Conflict of Interest

The authors declare that the research was conducted in the absence of any commercial or financial relationships that could be construed as a potential conflict of interest.
